# Exosomal miR-335 derived from mature dendritic cells enhanced mesenchymal stem cell-mediated bone regeneration of bone defects in athymic rats

**DOI:** 10.1186/s10020-021-00268-5

**Published:** 2021-02-26

**Authors:** Zhongliu Cao, Yanfeng Wu, Lingling Yu, Lingfeng Zou, Liu Yang, Sijian Lin, Jue Wang, Zhen Yuan, Jianghua Dai

**Affiliations:** Department of Rehabilitation Medicine, The Second Affiliated Hospital of Nanchang University, No. 1 Minde Road, Nanchang, 330006 China

**Keywords:** Mesenchymal stem cells, Dendritic cell, Exosome, miR-335, Bone defect

## Abstract

**Background:**

Transplantation of bone marrow-derived mesenchymal stem cells (BM-MSCs) embedded in a bio-compatible matrix has been demonstrated as a promising strategy for the treatment of bone defects. This study was designed to explore the effect and mechanism of exosomes derived from mature dendritic cells (mDC-Exo) on the BM-MSCs-mediated bone regeneration using the matrix support in an athymic rat model of femoral bone defect.

**Methods:**

The BM-MSCs were isolated from rats and incubated with osteoblast induction medium, exosomes derived from immature DCs (imDC-Exo), mDC-Exo, and miR-335-deficient mDC-Exo. BM-MSCs treated without or with mDC-Exo were embedded in a bio-compatible matrix (Orthoss^®^) and then implanted into the femoral bone defect of athymic rats.

**Results:**

mDC-Exo promoted the proliferation and osteogenic differentiation of BM-MSCs by transferring miR-335. Mechanistically, exosomal miR-335 inhibited Hippo signaling by targeting large tongue suppressor kinase 1 (LATS1) and thus promoted the proliferation and osteogenic differentiation of BM-MSCs. Animal experiments showed that mDC-Exo enhanced BM-MSCs-mediated bone regeneration after bone defect, and this effect was abrogated when miR-335 expression was inhibited in mDC-Exo.

**Conclusion:**

mDC-Exo promoted osteogenic differentiation of BM-MSCs and enhanced BM-MSCs-mediated bone regeneration after femoral bone defect in athymic rats by transferring miR-335.

## Background

Bone defects can be caused by trauma, bone tumors, infections, congenital bone defects, and other diseases. The treatment of bone defects has always been a major challenge to clinicians. At present, the gold standard treatment for bone defect is autologous bone graft. However, the amount of autogenous bone is limited, and complications such as bleeding, infection, pain, and fracture may occur in the donor site (Dimitriou et al. 2011). Tissue engineering through the use of bone-regenerating stem cells embedded in a bio-compatible matrix has been demonstrated as a promising strategy for the treatment of bone defects (Al-Moraissi et al. 2020; Li et al. 2018a).

Mesenchymal stem cells (MSCs) are multipotent stem cells with the capacity for self-renewal and multi-lineage differentiation that can be induced to differentiate into adipocytes, chondroblasts, osteoblasts, hepatocyte-like cells, and so on (Squillaro et al. 2016). Bone marrow-derived MSCs (BM-MSCs) seeded onto matrices have been shown to have bone-regenerating capacity in several animal models of bone defects (Kon et al. 2000; Hsieh et al. 2018). Among the various organic and inorganic osteoinductive matrices tested in vitro and in vivo, natural bone minerals (Orthoss^®^) have been proven to provide excellent, biocompatible matrices and also amenable to remodelling in the process of bone regeneration (Kim and Kim 2008). It has been shown that BM-MSCs seeded onto Orthoss^®^ and implanted into in athymic rats which underwent bone defect could induce new bone formation (Burastero et al. 2010).

Although the bone-regenerating effect of MSC has been confirmed, there are still some problems, such as unstable regeneration effect, difference in tissue structure between regeneration and natural bone, and so on. Convincing evidence indicates that optimizing the host’s immune microenvironment can help to enhance MSC-mediated bone-regenerating effect (Liu et al. 2011; Chen et al. 2014, 2015). Dendritic cells (DCs), particularly important innate immune cells, can promote MSC recruitment, enhance MSC migration, and induce osteogenic differentiation of MSCs through secreting exosomes (Silva et al. 2017; Zhang et al. 2017; Wang et al. 2014). Exosomes, the nanosized (30–150 nm) extracellular vesicles released from various cell types, have recently emerged as crucial mediators of intercellular communication by delivering various molecules (e. g. proteins, mRNA, and microRNAs) from parent to recipient cells (Cañas et al. 2019; Hessvik and Llorente 2018). Exosomes have been emerged as a promising therapeutic strategy in the field of bone tissue engineering (Li et al. 2018a).

The present study was designed to explore the effect and mechanism of exosomes derived from mature DCs (mDC-Exo) on the BM-MSCs-mediated bone regeneration using Orthoss^®^ as the matrix support in an athymic rat model of femoral bone defect. Our findings demonstrated that mDC-Exo enhanced BM-MSCs-mediated bone regeneration after bone defect by transferring miR-335.

## Materials and methods

### Preparation of imDCs and mDCs from rat bone marrow cells

Bone marrow cells were collected by flushing femurs and tibias of six SD rats (4-week-old, male or female). Bone marrow mononuclear cells were isolated from bone marrow using Ficoll-Paque density gradient centrifugation. Then, CD34^+^ hematopoietic Progenitor cells (HPC) were isolated from bone marrow mononuclear cells using the MACS Direct CD34 Progenitor Cell Isolation Kit and MiniMACS magnetic-based positive-selection system (Miltenyi Biotec) according to manufacturer’s protocol. The CD34^+^HPC were induced with 10 ng/ml recombinant granulocyte macrophage-colony stimulating factor (rGM-CSF) and 10 ng/ml recombinant interleukin 4 (rIL-4) in vitro for 7 days to generate immature DCs (imDCs). The mature DCs (mDCs) were generated by culturing imDCs with lipopolysaccharide (LPS, 1 μg/ml) for another 2 days.

### Isolation and identification of exosomes from imDCs and mDCs

The exosomes were isolated from the cell supernatant of imDCs and mDCs by multi-step centrifugation (Kim et al. 1950). Briefly, cell supernatant was obtained after centrifugation (300×*g* for 10 min, 1200×*g* for 20 min, and then 10,000×*g* for 30 min) to remove cell debris and microvesicles. The supernatant was then centrifuged at 100,000×*g* for 60 min. The exosome pellet was washed in PBS and then centrifuged at 100,000×*g* for 60 min. The exosome pellet was resuspended in PBS and passed through a 0.22 μm syringe filter. The amount of exosomes collected was measured by the BCA protein Kit Assay (Thermo Scientific, USA). The obtained exosomes were referred to as imDC-Exo and mDC-Exo, respectively.

The morphologic characteristics of exosomes were observed by transmission electron microscopy (TEM) (Hitachi, Japan) as previously described (Li et al. 2018b). The particle size of exosomes was then measured using a NanoSight NS500 (Malvern Instruments, UK). The imDCs and mDCs were lysed with cell lysis buffer. Exosomes were lysed using the Total Exosome RNA and Protein Isolation Kit. Cell lysates and exosome lysates were then subjected to western blot analysis to detect the protein levels of Calnexin, CD63, and Alix.

### Isolation of rat BM-MSCs

The BM-MSCs were isolated from the bone marrow of Sprague Dawley rats as previously described (Kolakshyapati et al. 2017). Briefly, bone marrow was collected by flushing femurs and tibias and cultured in complete medium (α-MEM) containing 10% fetal bovine serum (FBS; Gibco, USA) and antibiotics at 37 °C under 5% CO_2_. The rat BM-MSCs at the third passage were used in this study and prepared for surface markers detection according to the methods reported previously (Chen et al. 2019). The osteogenic and adipogenic differentiation capacity of BM-MSCs was examined by alizarin red and Oil Red O staining, respectively (Wenkai et al. 2016). Animal experiments were approved by the Ethics Committee of The Second Affiliated Hospital of Nanchang University.

### Co-culture of BM-MSCs and exosomes

The BM-MSCs at the third passage were plated in 24-well plates at a density of 1 × 10^4^ cells per well. The BM-MSCs were randomly divided into the following groups: (1) control: incubated with α-MEM medium; (2) Induction: incubated with osteoblast induction medium (α-MEM medium containing 10 mM β-glycerophosphate, 50 μM vitamin C, and 100 nM dexamethasone); (3) imDC-Exo: incubated with 10 μg/ml imDC-Exo and α-MEM medium; (4) mDC-Exo: incubated with 10 μg/ml mDC-Exo and α-MEM medium; (5) mDC-Exo-NC: incubated with 10 μg/ml mDC-Exo-NC and α-MEM medium; (6) mDC-Exo-miR-335I: incubated with 10 μg/ml mDC-Exo-miR-335I and α-MEM medium. mDCs were transfected with miR-335 inhibitors or inhibitor NC (GenePharma, Shanghai, China), and then exosomes were isolated from the transfected mDCs according to the methods described above, referred to as mDC-Exo-NC and mDC-Exo-miR-335I, respectively. The treated BM-MSCs were harvested on the 7th and 14th day of culture.

### Transfection in BM-MSCs

BM-MSCs were transfected with miR-335 mimics, mimic NC, miR-335 inhibitors, inhibitor NC, LATS1 (large tongue suppressor kinase 1) overexpression vector, and empty vector (GenePharma, Shanghai, China) using Lipofectamine 3000 (Invitrogen, USA) following the manufacturer’s instructions*.*

### 5-bromo-2-deoxyuridine (BrdU) staining

The effect of mDC-Exo on cell proliferation of BM-MSCs was assessed by measuring BrdU incorporation as previously described (Ferry et al. 2011). BM-MSCs were seeded in a 24-well plate at a density of 1 × 10^4^ cells/well and then incubated with imDC-Exo or mDC-Exo for the desired time at the same time as BrdU co-incubation was performed using the BrdU Staining Kit (Invitrogen, USA). BrdU-stained cells were washed and then stained with Hoechst 33342 for nuclear staining. Images were taken using a fluorescence microscope. The percentage of BrdU-positive cells to total cell number was calculated.

### Determination of alkaline phosphatase (ALP) activity

ALP activity was measured using an Alkaline Phosphatase Activity Detection kit (Nanjing Jiancheng Bioengineering Institute, Nanjing, China) at 520 nm with a spectrophotometer according to the manufacturer’s instructions.

### Real-time quantitative PCR (qRT-PCR)

Total RNA was extracted using TRIzol (Invitrogen, USA) and miRNA was extracted using the miRNeasy kit (Qiagen, Germany). Then, RNA was reverse-transcribed to cDNA using a Transcriptor First Strand cDNA synthesis kit (Roche, USA). The expression levels of miR-672, miR-335, miR-124, and miR-125a-5p were detected using the miRNA qRT-PCR kit (GeneCopoeia) whereas the expression of Runx2 was using the SYBR premix (Takara, Dalian) in Applied Biosystems 7500 PCR system (Foster, CA). The U6 and GAPDH were used as the internal controls for miRNA and mRNA, respectively. The relative expression of genes was analyzed using 2^−∆∆CT^ method.

### Luciferase activity assay

The binding sites of miR-335 on the 3′-UTR of LATS1 were identified using TargetScan online bioinformatics software (http://www.targetscan.org) and were verified by the luciferase reporter assay. LATS1 3′-UTR was amplified by PCR and then cloned into a pGL3 vector (Promega, USA). HEK293T cells were co-transfected with wide-type (WT) or mutant (Mut) LATS1 3′-UTR luciferase reporter plasmids and miR-335 mimic or mimic NC using Lipofectamine 2000 reagent (Invitrogen, USA). The luciferase activity was determined using the Dual-Luciferase Reporter Assay System Kit (Promega, USA).

### Preparation of the graft

For the preparation of graft implants, BM-MSCs at the third passage were incubated without or with mDC-Exo, mDC-Exo-NC, or mDC-Exo-miR-335I at 37 °C with 5% CO_2_ as described above. After one week, untreated or exosome-treated BM-MSCs (2 × 10^6^) were centrifuged at 400×*g* for 5 min and then resuspended in of PBS (100 µl) containing 10 mM glucose. The cell suspension was mixed with a matrix (Orthoss®; Geistlich Biomaterials, Wolhusen, Switzerland), with 200 µl of 2% carboxymethyl cellulose-based hydrogel (Sigma, USA).

### Establishment of a rat model of bone defect and graft implantation

A total of 15 male athymic rats were purchased and maintained under specific pathogen-free conditions (temperature: 24 ± 2 °C; humidity: 55%; a 12 h/12 h light/dark cycle) and were fed a normal rodent diet. All animal experiments were performed following the guidelines for Laboratory Animal Care and Use of The Second Affiliated Hospital of Nanchang University.

A rat model of bone defect was established according to a previous study (Burastero et al. 2010). Rats were anesthetized with diazepam (1 mg/100 g body weight; i.p.), Xylazine (0.5 mg/100 g body weight; i.m.), and Ketamine (4 mg/100 g body weight; i.m.). The right hind limb of male athymic rats was shaved and prepared. The anterior surface of the right femur was exposed from the anterolateral side, saving muscles and tendons. Before femoral osteotomy, a polymethyl-methacrylate plate (3-mm thick and 25-mm long) was fixed with four stainless steel cerclage wires. Then, a femoral gap of 6 mm was cut in the central diaphysis and filled with the prepared graft. After transplantation, all the nude rats were free to eat water. The immune response of rats after transplantation was observed at any time. Body weight, mental state, feeding activity and nutritional status were observed every 2 days.

### Micro-computed tomography (micro-CT) scan

At weeks 4, 8, and 12 following surgery, rats were sacrificed and the operated femurs were harvested, dissected free of the surrounding muscle tissue, and fixed in 10% formaldehyde in PBS. The specimens were scanned using Aloka Latheta LCT-200 micro-CT (Hitachi, Japan). The scanning parameters were as follows: 80 kV voltage, 0.5 mA current, 48 μm layer thickness, and 48 μm resolution. Three-dimensional reconstructions were performed using Mimics16.0. The volume of new bone was calculated.

### Hematoxylin & eosin (H&E) staining

The operated femurs of the right hind limb of rats were fixed in 10% neutral formaldehyde for 48 h at 4 °C and decalcified in 10% EDTA solution at 4 °C for 4 weeks. After dehydrated, tissues were embedded in paraffin and cut into 5-μm sections. The sections were stained with H&E kit (Sigma-Aldrich, USA) to evaluate the growth of new bone in the bone defect area following the manufacturer’s instructions.

### Western blot

Total proteins were extracted from exosomes, whole-cell lysates, or homogenized femur tissue using the radioimmunoprecipitation assay lysis buffer (Beyotime, Haimen, China). Then western blot was performed according to a previous study (Guo et al. 2020). The primary antibodies against Alix, CD63, Calnexin, ALP, runt-related transcription factor 2 (Runx2), large tumor suppressor 1 (LATS1), and GAPDH were purchased from Abcam (1:1000). The primary antibodies against YAP, phosphor (p)-YAP, p-LAST1, TAZ, and p-TAZ were purchased from Cell Signaling Technology (1:1000). The signals were visualized using an ECL chemiluminescent system (Pierce, USA).

### Quantification of western blots

Bands were semi-quantified by image intensity under the curve. The protein levels were calculated by the ratio of the protein to GAPDH. In our study, the data was presented as bar graphs, the control samples (or Matrix group sample in vivo) were set at 1, and the protein level displayed fold change in intensity. Data are representative of at least three independent experiments.

### Statistical analysis

All statistical analyses were conducted using GraphPad Prism 7.0. A statistical comparison of different treatment groups was determined by one-way analysis of variance followed by Tukey’s post-test. P values < 0.05 were considered significant.

## Results

### Characterization of exosomes

**Fig. 1:**
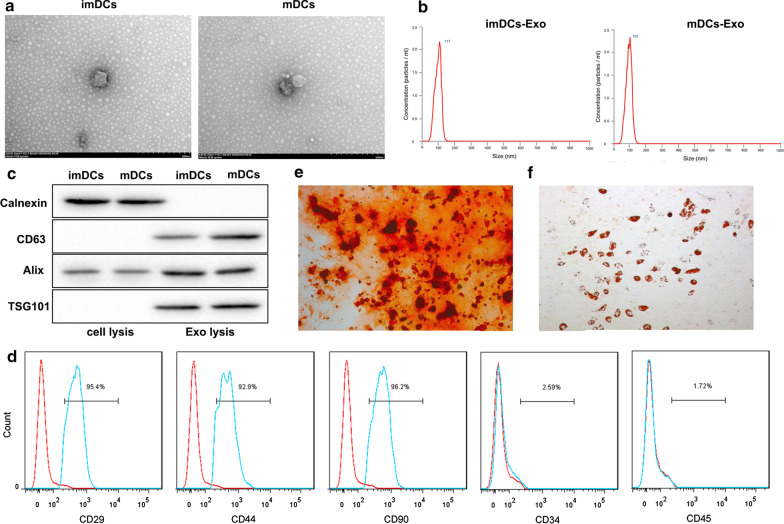
Identification of exosomes and BM-MSCs. The exosomes isolated from imDCs and mDCs were referred to as imDCs-Exo and mDCs-Exo, respectively. **a** The morphological characterization of imDCs-Exo and mDCs-Exo by TEM. **b** The particle size of imDCs-Exo and mDCs-Exo measured using nanoparticle tracking analysis. **c** Expression of the exosomal markers (CD63, and Alix) and the exosome negative marker (Calnexin) confirmed by western blot. **d** The surface antigens including CD29, CD44, CD90, CD34, and CD45 of rat BM-MSCs was detected by flow cytometry. The positive rate of each surface marker was presented. **e** Osteogenic differentiation of BM-MSCs was confirmed by Alizarin Red staining. **f** Adipogenic differentiation of BM-MSCs was identified using Oil Red O staining

The exosomes derived from imDCs and mDCs were characterized by TEM, nanoparticle tracking analysis, and western blot analyses. The isolated vesicles were spherical with a size distribution between 0 and 150 nm in diameter (Fig. 1a, b), consistent with previously reported characteristics of exosomes (Jia et al. 2014). Furthermore, western blot confirmed that these nanoparticles expressed exosomal markers (Alix, CD63 and TSG101) but not the exosome negative marker (Calnexin) (Fig. 1c). These results confirmed that these nanoparticles were indeed exosomes.

### Characterization of BM-MSCs

The rat BM-MSCs at the third passage were prepared for surface markers detection and multilineage differentiation capacity analysis. Flow cytometry showed the phenotypes of purified BM-MSCs were positive for CD29, CD44, and CD90, but negative for CD34 and CD45 (Fig. 1d), which were consistent with the characteristics of MSCs. Furthermore, we observed the potential of adipogenic and osteogenic differentiation of the obtained BM-BMSCs, as evidenced by calcium deposits stained by Alizarin Red and intracytoplasmic lipid droplets stained by Oil Red O (Fig. 1e, f).

### mDC-Exo promoted proliferation and osteogenic differentiation of BM-MSCs

**Fig. 2:**
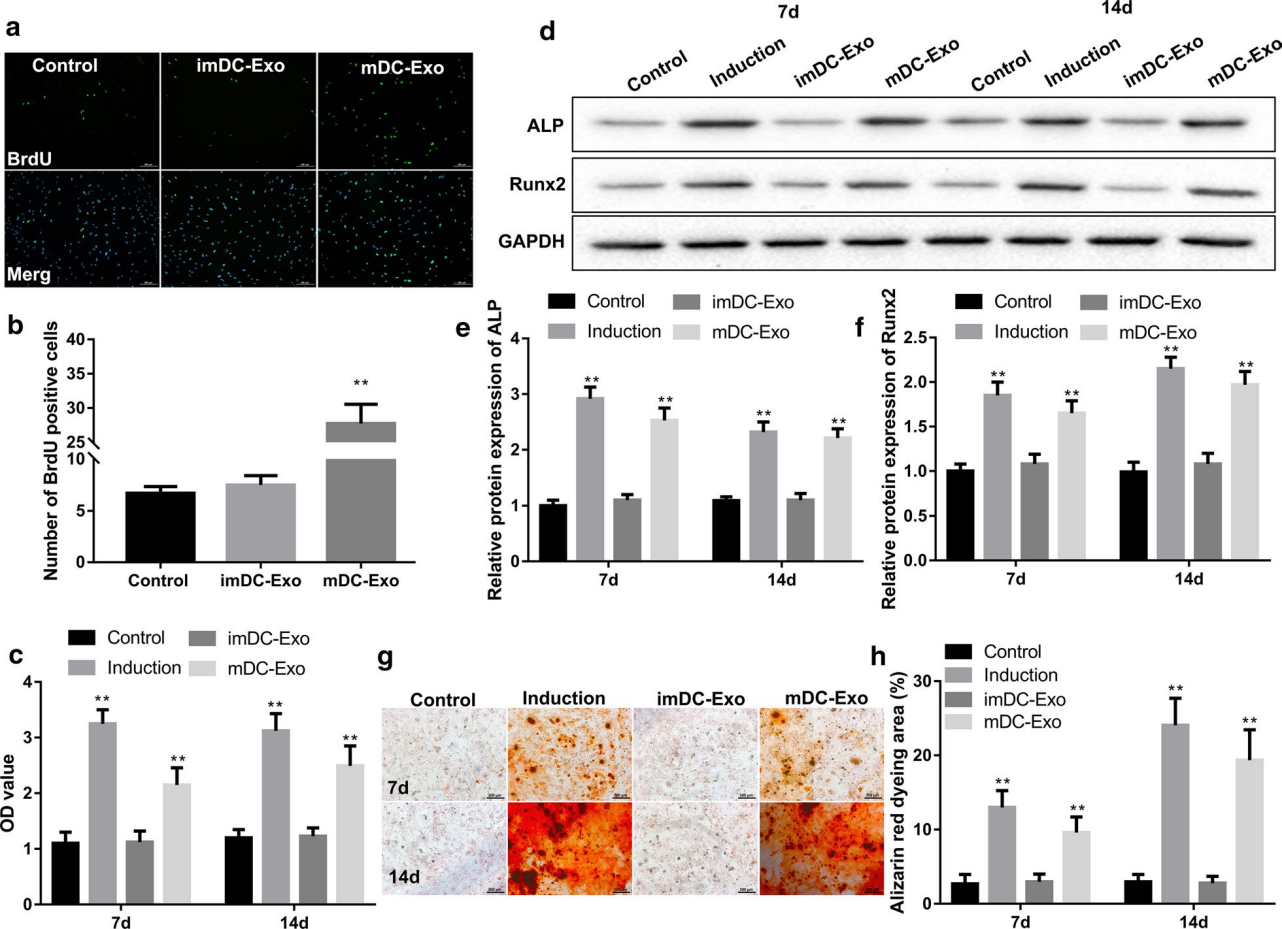
mDC-Exo promoted proliferation and osteogenic differentiation of BM-MSCs. **a** Immunofluorocytochemical staining for BrdU (green) and DNA (DAPI, blue) in BM-MSCs and **b** quantitation of the number of BrdU-positive cells in BM-MSCs in the groups of control, imDC-Exo, and mDC-Exo. **c** ALP activities expressed in optical density (OD) values at 520 nm, **d–f** protein levels of ALP and Runx2 determined by western blot, **g**, **h** representative Alizarin red staining assessing calcium deposits and quantification of the percentage of Alizarin red dyeing area in BM-MSCs in the groups of control, induction, imDC-Exo, and mDC-Exo on the 7th and 14th day of culture. **P < 0.01, vs. Control

We examined the effects of imDC-Exo and mDC-Exo on the proliferative capacity of BM-MSCs by BrdU staining. The percentage of BrdU-positive cells was significantly increased in mDC-Exo-incubated BM-MSCs when compared with the control medium-incubated BM-MSCs. However, incubation with imDC-Exo had no obvious effect on the proliferative capacity of BM-MSCs (Fig. 2a, b). To further clarify the effects of imDC-Exo and mDC-Exo on the capacity of osteogenic differentiation of BM-MSCs, we detected expression of osteogenic differentiation markers (ALP and Runx2) and performed Alizarin red staining. Expectedly, incubation with osteoblast induction medium markedly increased activity of ALP (Fig. 2c) and protein levels of ALP and Runx2 (Fig. 2d–f), and promoted calcium deposition (Fig. 2g–h) in BM-MSCs on the 7th and 14th day of culture. Similarly, incubation with mDC-Exo also significantly facilitated the osteogenic differentiation capacity of BM-MSCs, as evidenced by increases in expression of ALP and Runx2 and percentage of Alizarin red dyeing area (Fig. 2c–h). However, incubation with imDC-Exo had no obvious effect on the osteogenic differentiation of BM-MSCs (Fig. 2c–h).

### mDC-Exo promoted osteogenic differentiation of BM-MSCs by transferring miR-335

**Fig. 3:**
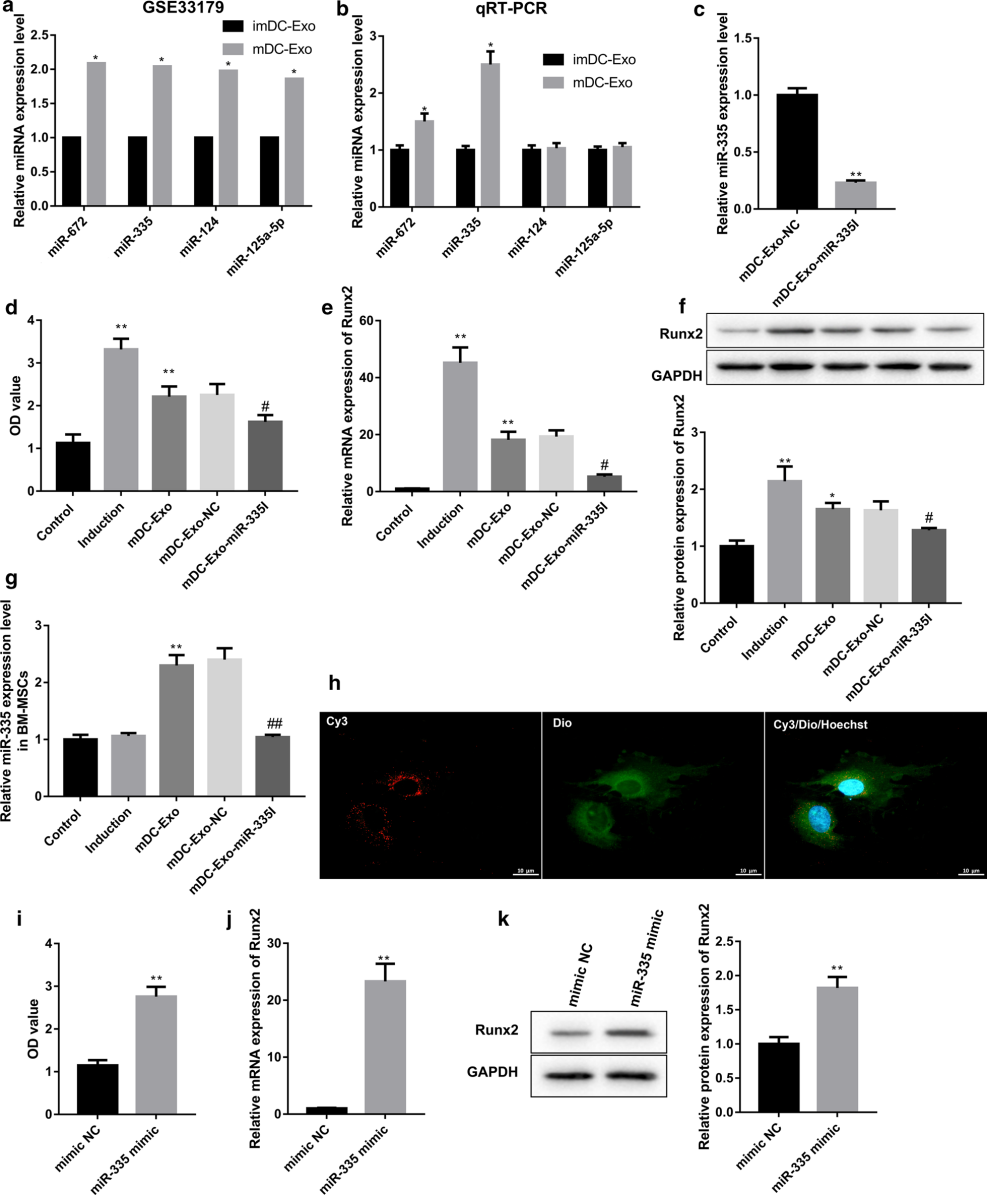
mDC-Exo promoted osteogenic differentiation of BM-MSCs by transferring miR-335. **a** Higher expression of miR-672, miR-335, miR-124, and miR-125a-5p in mouse mDC-Exo relative to imDC-Exo (GSE33179). *P < 0.05, vs. imDC-Exo. **b** Expression of miR-672, miR-335, miR-124, and miR-125a-5p determined by qRT-PCR analysis in imDC-Exo and mDC-Exo separated according to the method mentioned above. *P < 0.05, vs. imDC-Exo. **c** miR-335 expression determined by qRT-PCR analysis in mDC-Exo-NC and mDC-Exo-miR-335I. **P < 0.01, vs. mDC-Exo-NC. **d** ALP activities expressed in optical density (OD) values at 520 nm, **e**, **f** Runx2 mRNA and protein levels, and **g** miR-335 expression in BM-MSCs in the groups of control, induction, mDC-Exo, and mDC-Exo-NC, and mDC-Exo-miR-335I on the 7th day of culture. *P < 0.05, **P < 0.01, vs. Control; ^#^P < 0.05, ^##^P < 0.01, vs. mDC-Exo-NC. **h** The exosomes isolated from Cy3 (red)-miR-335-transfected mDCs were labeled with the lipophilic fluorescent dye Dio (green) and incubated with BM-MSCs (nuclei stained with Hoechst 33,342, blue). The uptake of exosomes by BM-MSCs was observed by confocal laser microscopy. **(I)** ALP activities expressed in OD values at 520 nm, **(J-K)** Runx2 mRNA and protein levels in BM-MSCs transfected with miR-335 mimics and mimic NC. **P < 0.01, vs. mimic NC

Next, we sought to determine the molecular mechanism by which mDC-Exo promoted osteogenic differentiation of BM-MSCs. Data from the GEO database (GSE33179) showed that miRNAs, including miR-672, miR-335, miR-124, and miR-125a-5p, were highly expressed in mouse mDC-Exo when compared with the mouse imDC-Exo (Montecalvo et al. 2012) (Fig. 3a). To explore whether mDC-Exo promoted osteogenic differentiation of BM-MSCs by delivering these miRNAs, we detected expression of these miRNAs in imDC-Exo and mDC-Exo. We found that among these four miRNAs, miR-672 and miR-335 showed significantly higher levels in mDC-Exo, especially for miR-335 (Fig. 3b). To clarify the contribution of exosomal miR-335 in the osteogenic differentiation-promoting effect of mDC-Exo, we developed miR-335-deficient mDC-Exo (mDC-Exo-miR-335I) by transfecting mDCs with miR-335 inhibitors followed by exosome isolation. qRT-PCR analysis confirmed that miR-335 expression was much lower in mDC-Exo-miR-335I than that in mDC-Exo-NC (Fig. 3c). We then treated BM-MSCs with mDC-Exo, mDC-Exo-NC, and mDC-Exo-miR-335I. Incubation with mDC-Exo notably increased ALP activity (Fig. 3d) and Runx2 mRNA and protein levels (Fig. 3e, f). Furthermore, mDC-Exo incubation led to a remarkable increase in miR-335 expression in BM-MSCs (Fig. 3g). However, the promoting effects of mDC-Exo on BM-MSCs osteogenic differentiation were compromised when miR-335 expression in mDC-Exo was inhibited (Fig. 3d–g). Moreover, we labeled the exosomes from Cy3-miR-335-transfected mDCs with Dio and then added to BM-MSCs. We observed exosomal transferred miR-335 (red signals) in the cytoplasm of BM-MSCs, which confirmed the uptake of exosomes by BM-MSCs (Fig. 3h). Therefore, these results indicated that mDC-Exo promoted osteogenic differentiation of BM-MSCs by transferring miR-335.

### miR-335 overexpression promoted osteogenic differentiation of BM-MSCs

To further confirm the promoting effect of miR-335 on BM-MSCs osteogenic differentiation, we overexpressed miR-335 by transfecting BM-MSCs with miR-335 mimics. The results showed that the ALP activity (Fig. 3i) and Runx2 mRNA and protein levels (Fig. 3j, k) were markedly increased in BM-MSCs following transfection with miR-335 mimics.

### Exosomal miR-335 decreased LATS1 expression in BM-MSCs by targeting LATS1

**Fig. 4:**
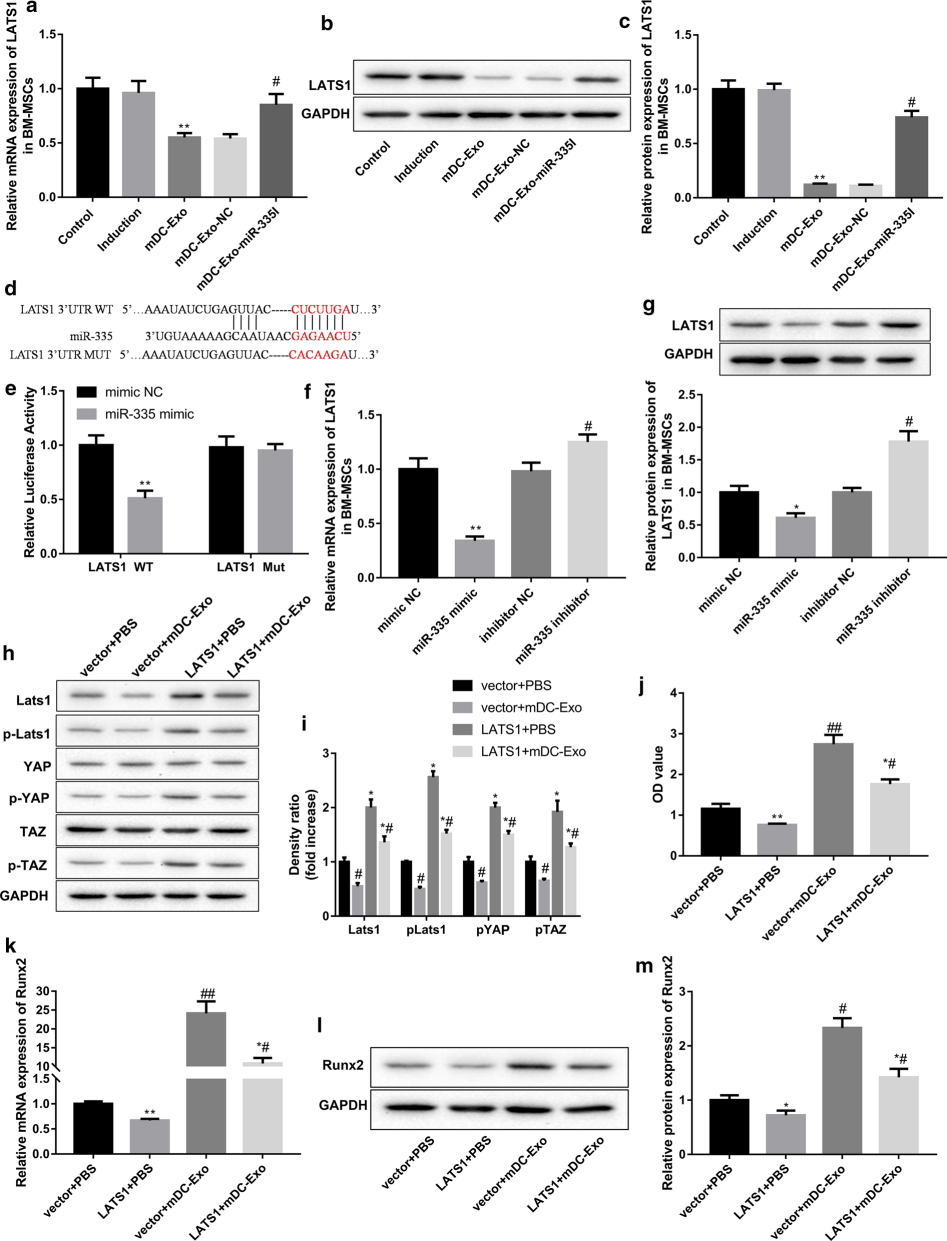
Exosomal miR-335 promoted osteogenic differentiation of BM-MSCs by targeting LATS1. LATS1 mRNA (**a**) and protein (**b, c**) levels in BM-MSCs in the groups of control, induction, mDC-Exo, and mDC-Exo-NC, and mDC-Exo-miR-335I on the 7th day of culture. **P < 0.01, vs. Control; ^#^P < 0.05, vs. mDC-Exo-NC. **d** Predicted binding sites for miR-335 in the 3′-UTR of LATS1 (Targetscan). **e** The interaction between miR-335 and LAST1 3′-UTR was analyzed by luciferase activity assay. **P < 0.01, vs. mimic NC. LAST1 mRNA (**f**) and protein (**g**) levels in BM-MSCs transfected with miR-335 mimics, mimic NC, miR-335 inhibitors, or inhibitor NC. *P < 0.05, **P < 0.01, vs. mimic NC; ^#^P < 0.05, vs. inhibitor NC. **h**, **i** The protein levels of LATS1, p-LAST1, YAP, p-YAP, TAZ, and p-TAZ, **j** ALP activities expressed in optical density (OD) values at 520 nm, **k–m** Runx2 mRNA and protein levels in BM-MSCs transfected with LAST1 overexpression vector/empty vector and treated with mDC-Exo or PBS. *P < 0.05, **P < 0.01, vs. vector + PBS; ^#^P < 0.05, ^##^P < 0.01, vs. vector + mDC-Exo

To explore the molecular mechanisms underlying the osteoinductive capacity of miR-335, we sought to identify the putative miR-335 targets using the target prediction programs TargetScan and found that LATS1 is a putative target of miR-335 (Fig. 4d). LATS1 is a key component of the Hippo signaling that is involved in regulating the osteogenic differentiation of MSCs (An et al. 2019). We found that LATS1 expression in BM-MSCs was reduced following incubation with mDC-Exo. However, the suppressive effect of mDC-Exo on LATS1 expression was abated when miR-335 expression was inhibited (Fig. 4a–c). Luciferase activity assay showed that miR-335 mimic transfection significantly decreased luciferase activity in the LATS1 WT group, whereas had no obvious effect on that in the LATS1 Mut group, which confirmed that LATS1 was a direct target of miR-335 (Fig. 4e). Furthermore, transfection with miR-335 mimics notably decreased LATS1 expression in BM-MSCs, whereas transfection with miR-335 inhibitors yielded the opposite results (Fig. 4f, g).

### Exosomal miR-335 inhibited Hippo signaling and promoted osteogenic differentiation of BM-MSCs by targeting LATS1

When the Hippo signaling is activated, MST1/2 (mammalian Ste20-like kinases) phosphorylate LATS1/2 kinases, and then LATS1/2 directly phosphorylate and inhibit the activities of YAP and TAZ, leading to inhibition of osteogenic-specific genes including Runx2 (Wang et al. 2018; Tang et al. 2016). Western blot analyses revealed that incubation with mDC-Exo inhibited Hippo signaling in BM-MSCs, as evidenced by decreases in expression of LATS1, p-LATS1, p-YAP, and p-TAZ. In contrast, upregulation of LATS1 expression by LATS1 overexpression activated Hippo signaling and abrogated the mDC-Exo-mediated inhibitory effects on Hippo signaling (Fig. 4h, i). Importantly, the promoting effects of mDC-Exo on ALP activity (Fig. 4j) and Runx2 expression were partially abolished by LATS1 overexpression (Fig. 4k–m). These results collectively indicated that exosomal miR-335 targeted LATS1, leading to inhibition of Hippo signaling and subsequent promotion of BM-MSCs differentiation into osteoblasts.

### mDC-Exo enhanced BM-MSCs-mediated bone regeneration after bone defect by transferring miR-335

**Fig. 5:**
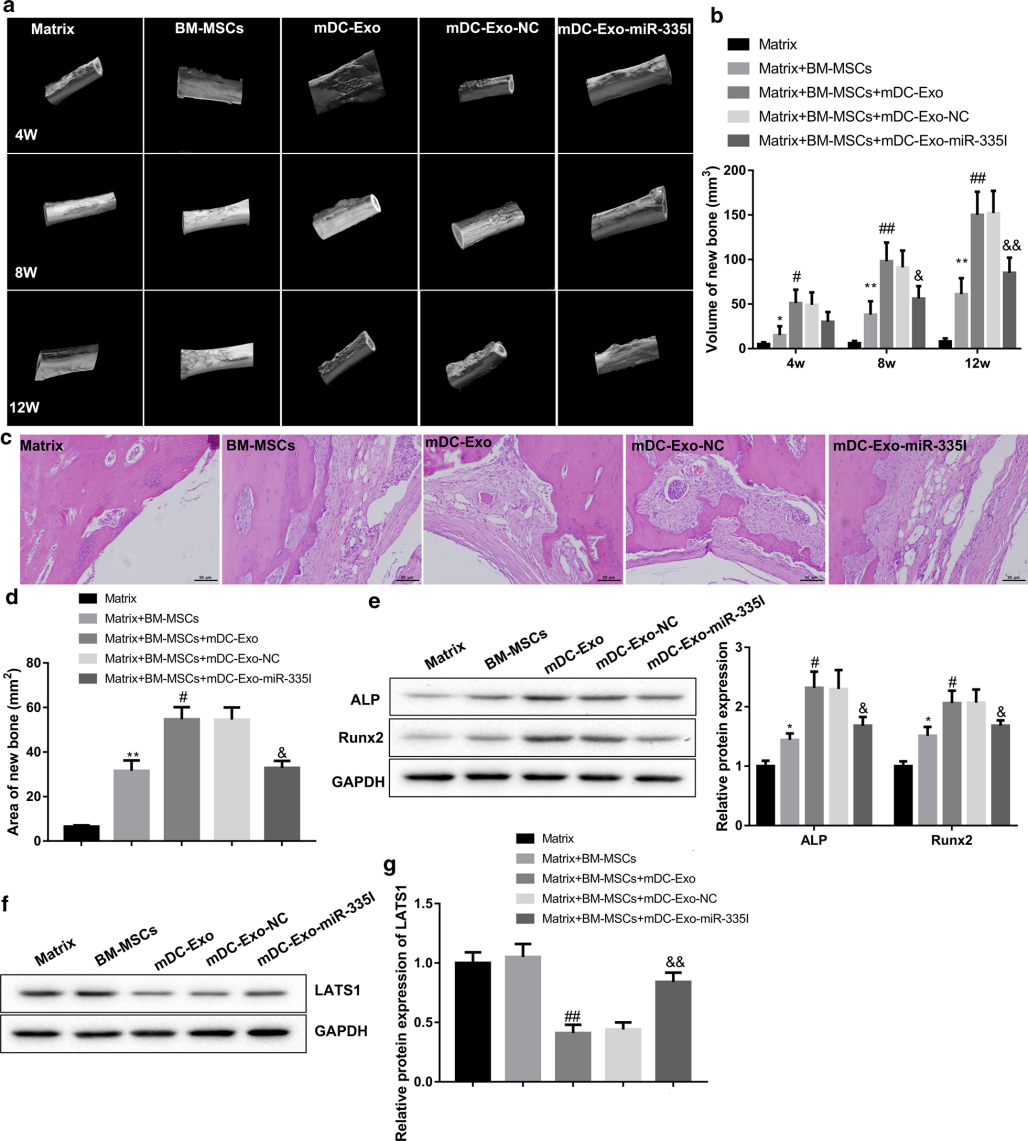
mDC-Exo enhanced BM-MSCs-mediated bone regeneration by transferring miR-335. Rats were randomly divided into five groups: Matrix, Matrix + BM-MSCs, Matrix + BM-MSCs + mDC-Exo, Matrix + BM-MSCs + mDC-Exo-NC, and Matrix + BM-MSCs + mDC-Exo-miR-335I. **a** Bone regeneration was monitored via micro-CT scanning at 4, 8 and 12 weeks post-surgery. **b** Quantification of new bone volume from micro-CT scanning. **c** Representative H&E staining showing bone defect repair of posterior limbs of rats. **d** Quantification of new bone area from H&E staining. The protein levels of **e** ALP, Runx2, and **f**, **g** LAST1 in the bone examined by western blot. *P < 0.05, **P < 0.01, vs. Matrix; ^#^P < 0.05, ^##^P < 0.01, vs. Matrix + BM-MSCs; ^$^P < 0.05, ^$$^P < 0.01, vs. Matrix + BM-MSCs + mDC-Exo-NC

Finally, we explored the in vivo effect of mDC-Exo on the BM-MSCs-mediated bone regeneration in an athymic rat model of femoral bone defect. BM-MSCs treated without or with mDC-Exo were embedded in a bio-compatible matrix (Orthoss®) to prepare the grafts. The prepared grafts were implanted into the bone defect. Micro-CT scanning and three-dimensional reconstruction observation showed that in the Matrix group, the bone defect area had nearly no evidence of new bone formation. In the Matrix + BM-MSCs group, the volume of new bone tissues in the bone defect area was increased. In the Matrix + BM-MSCs + mDC-Exo group and the Matrix + BM-MSCs + mDC-Exo-NC group, the bone growth in the defect was further facilitated at each time point post-surgery. However, the osteogenic effect of BM-MSCs-mDC-Exo was compromised when miR-335 expression was inhibited in mDC-Exo (Fig. 5a, b). H&E staining evaluating bone defect repair of posterior limbs of rats showed that the area of new bone was increased in the Matrix + BM-MSCs group when compared with the Matrix group and further increased in the Matrix + BM-MSCs + mDC-Exo group and the Matrix + BM-MSCs + mDC-Exo-NC group. However, the area of new bone was decreased in the Matrix + BM-MSCs + mDC-Exo-miR-335I group as compared to the Matrix + BM-MSCs + mDC-Exo-NC group (Fig. 5c, d). Consistently, the protein levels of ALP and Runx2 in the bone were significantly higher in the Matrix + BM-MSCs + mDC-Exo group when compared with the Matrix + BM-MSCs group. However, when compared with the Matrix + BM-MSCs + mDC-Exo-NC group, the levels of ALP and Runx2 were decreased in the Matrix + BM-MSCs + mDC-Exo-miR-335I group (Fig. 5e, f). Moreover, LATS1 protein level was decreased in the Matrix + BM-MSCs + mDC-Exo group but was increased in the Matrix + BM-MSCs + mDC-Exo-miR-335I group (Fig. 5g).

## Discussion

Extensive evidence suggests that BM-MSCs have osteoinductive capacities and are biologically safe in various experimental animal models of bone defect (Kon et al. 2000; Hsieh et al. 2018; Kim and Kim 2008). A large number of studies have shown that immune cells (macrophages, T cells and B cells, etc.) can affect MSCs mediated bone regeneration, and optimizing the host's immune microenvironment can promote stem cell-based bone regeneration(Tee and Sun 2020; Kovach et al. 2015). Moreover, studies have confirmed that MSCs can regulate a variety of immune cell functions, including macrophages, T cells and B cells, NK cells and DCs (Hu et al. 2020), among which the regulation of DCs may be the main target of MSC immune regulation. The functional regulation between cells and cells is mutual, and the regulated DCs may also reverse regulate MSCs function through unknown pathways. It has also been demonstrated that DCs can promote MSC recruitment, enhance MSC migration, and induce osteogenic differentiation of MSCs through paracrine cytokines or exosomes (Silva et al. 2017; Zhang et al. 2017; Wang et al. 2014), therefore, we speculated that DCs may play an important role in fracture healing by affecting the osteogenic differentiation of MSC.

As a biocompatible matrix, orthoss is used to fill the large femoral gap and provide growth and differentiation support for host cells and donor cells (Burastero et al. 2010). In the study, we explored the effect of mDC-Exo on the BM-MSCs-mediated bone regeneration after bone defect using Orthoss® as the matrix support in an athymic rat model of femoral bone defect. To avoid rejection of the transplanted cells, the male athymic rats were used. Our results showed that the bone defect was significantly alleviated with higher percentage of new bone formation in the athymic rats receiving the matrix and in vitro-expanded BM-MSCs in the presence of mDC-Exo when compared with those receiving the matrix and in vitro-expanded BM-MSCs without mDC-Exo. These findings indicated that mDC-Exo enhanced BM-MSCs-mediated bone regeneration after bone defect.

DCs are specialized antigen-presenting cells that exhibit phagocytic ability and suppress T-cell responses in their immature state, and exhibit immunostimulatory capacity and promote immunity in their mature state. In this study, we compared the effect of imDCs- and mDCs- derived exosomes on the osteogenic differentiation of BM-MSCs. We found that incubation with mDC-Exo promoted proliferation and osteogenic differentiation of BM-MSCs; however, imDC-Exo had no obvious effect on the proliferation and osteogenic differentiation of BM-MSCs. Then, we set out to determine the possible mechanisms of this difference. Exosomes derived from DCs can function through the delivery of various cargos, such as proteins, mRNA, and microRNAs. Montecalvo et al. (2012) reported that DCs release exosomes with different microRNA cargos depending on the maturation stage of the DCs. This suggests that the difference in the regulatory effects of mDC-Exo and imDC-Exo on the osteogenic differentiation of BM-MSCs may be due to the different miRNAs they carry. The study reported by Montecalvo et al. (2012) also showed that when compared with the mouse imDC-Exo, mouse mDC-Exo contained higher expression of a series of microRNAs. Among these upregulated expressed miRNAs, four miRNAs (miR-672, miR-335, miR-124, and miR-125a-5p) were selected for further validation in rat imDC-Exo and mDC-Exo in this study. The results from the qRT-PCR analysis confirmed that miR-335 expression was the highest in mDC-Exo among these four miRNAs, which suggested that mDC-Exo may promote the proliferation and osteogenic differentiation of BM-MSCs by delivering miR-335. Expectedly, the promoting effects of mDC-Exo on the BM-MSCs differentiation into osteoblasts in vitro were abrogated when miR-335 expression was inhibited in mDC-Exo. Further animal studies also confirmed that inhibition of miR-335 expression attenuated the enhancement effect of mDC-Exo on the BM-MSCs-mediated bone regeneration after bone defect in athymic rats. Therefore, these results collectively demonstrated that mDC-Exo promoted osteogenic differentiation of BM-MSCs and enhanced BM-MSCs-mediated bone regeneration after bone defect by transferring miR-335.

Hippo signaling plays an important role in regulating the osteogenic differentiation of MSCs (Si et al. 2017). When the Hippo signaling is activated, MST1/2 phosphorylate LATS1/2 kinases, and then LATS1/2 directly phosphorylate YAP and TAZ. Phosphorylated YAP/TAZ is sequestered in the cytoplasm and cannot translocate to the nucleus to activate target gene transcription, leading to inhibition of osteogenic-specific genes including Runx2 (Wang et al. 2018; Tang et al. 2016). Therefore, activation of Hippo signaling is associated with inhibition of MSC osteogenic differentiation. Supporting this, An et al. (An et al. 2019) reported that GNAS knockdown suppresses osteogenic differentiation of MSCs via activation of Hippo signaling pathway. In this study, the online bioinformatics analysis combination with luciferase activity assays confirmed that LATS1 was a direct target of miR-335. Furthermore, we found that mDC-Exo, which delivered high amount of miR-335, inhibited Hippo signaling through downregulating LATS1 expression and subsequently promoted BM-MSCs differentiation into osteoblasts.

## Conclusions

In conclusion, this study demonstrates for the first time that mDC-Exo enhanced BM-MSCs-mediated bone regeneration after bone defect. Mechanistically, mDC-Exo inhibited LATS1 expression by transferring miR-335, and then inhibited Hippo signaling and subsequently promoted BM-MSCs differentiation into osteoblasts. Our findings provide a new idea for the treatment of bone defect by using bone tissue engineering in the future.

## Data Availability

The datasets used and/or analysed during the current study are available from the corresponding author on reasonable request.
